# HSP70-mediated neuroprotection by combined treatment of valproic acid with hypothermia in a rat asphyxial cardiac arrest model

**DOI:** 10.1371/journal.pone.0253328

**Published:** 2021-06-17

**Authors:** Joo Suk Oh, Jungtaek Park, Kiwook Kim, Hyun Ho Jeong, Young Min Oh, Semin Choi, Kyoung Ho Choi

**Affiliations:** Department of Emergency Medicine, Uijeongbu St. Mary’s Hospital, College of Medicine, The Catholic University of Korea, Uijeongbu-si, Republic of Korea; Universita degli Studi di Napoli Federico II, ITALY

## Abstract

It has been reported that valproic acid (VPA) combined with therapeutic hypothermia can improve survival and neurologic outcomes in a rat asphyxial cardiac arrest model. However, neuroprotective mechanisms of such combined treatment of valproic acid with hypothermia remains unclear. We hypothesized that epigenetic regulation of HSP70 by histone acetylation could increase HSP70-mediated neuroprotection suppressed under hypothermia. Male Sprague-Dawley rats that achieved return of spontaneous circulation (ROSC) from asphyxial cardiac arrest were randomized to four groups: normothermia (37°C ± 1°C), hypothermia (33°C ± 1°C), normothermia + VPA (300 mg/kg IV initiated 5 minutes post-ROSC and infused over 20 min), and hypothermia + VPA. Three hours after ROSC, acetyl-histone H3 was highly expressed in VPA-administered groups (normothermia + VPA, hypothermia + VPA). Four hours after ROSC, HSP70 mRNA expression levels were significantly higher in normothermic groups (normothermia, normothermia + VPA) than in hypothermic groups (hypothermia, hypothermia + VPA). The hypothermia + VPA group showed significantly higher HSP70 mRNA expression than the hypothermia group. Similarly, at five hours after ROSC, HSP70 protein levels were significantly higher in normothermic groups than in hypothermic groups. HSP70 levels were significantly higher in the hypothermia + VPA group than in the hypothermia group. Only the hypothermia + VPA group showed significantly attenuated cleaved caspase-9 levels than the normothermia group. Hypothermia can attenuate the expression of HSP70 at transcriptional level. However, VPA administration can induce hyperacetylation of histone H3, leading to epigenetic transcriptional activation of HSP70 even in a hypothermic status. Combining VPA treatment with hypothermia may compensate for reduced activation of HSP70-mediated anti-apoptotic pathway.

## Introduction

Although hypothermic targeted temperature management (TTM) has been introduced, post-cardiac arrest syndrome (PCAS) still shows high mortality and poor outcome. Many studies have investigated pharmacologic therapies to improve PCAS outcome. However, additive treatments that can enhance therapeutic effects of hypothermic TTM in clinical studies have not been reported. Valproic acid (VPA) is one candidate drug for preventing neuronal injury after cardiac arrest [1–3]. In addition to its antiepileptic property, VPA has neuroprotective effects by transcriptional activation of anti-apoptotic factors [4]. VPA has histone deacetylase (HDAC) inhibitor activity. It causes hyperacetylation of histone H3 and H4 [5]. Acetylation of histone leads to a more opened chromatin conformation, thus facilitating transcription of target genes. Heat shock protein 70 (HSP70), a well-known anti-apoptotic protein, is one target protein upregulated by VPA [6]. Two animal studies have reported that combination treatment of VPA and hypothermia improves survival and neurologic outcomes compared to hypothermia alone [2, 3]. However, it is unclear whether additive or synergistic neuroprotective signaling mechanism is involved. Current evidence does not support that HSP70 contributes to hypothermia-induced neuroprotection [7–11]. On the contrary, hypothermia can reduce the expression of HSP70, leading to attenuation of HSP70-mediated anti-apoptotic signaling pathways [7–13]. Therefore, we hypothesized that hypothermia would suppress HSP70-mediated neuroprotection and that such suppression could be compensated by VPA through epigenetic upregulation of HSP70. The aim of the present study was to investigate contribution of HSP70-mediated neuroprotection to enhanced neuroprotection offered by combined treatment of VPA with hypothermia.

## Materials and methods

This study was approved by Institutional Animal Care and Use Committee of the Catholic University of Korea (Protocol Number: UJA2017-17A). All surgery was performed under isoflurane anesthesia, and all efforts were made to minimize suffering.

### Animal preparation

Male Sprague-Dawley rats were provided free access to food and water *ad libitum* and held at controlled conditions with a temperature of 22°C ± 1°C and a 12 h/12 h of light/dark cycle for 5 days prior to experiment. Rats weighing 300–350 g at the time of experiment were anesthetized with 5% isoflurane using an insufflation chamber and then maintained with 2–3% isoflurane via facemask. The fur of each rat’s whole body including scalp was clipped to facilitate TTM and surgical procedures. After orotracheal intubation, mechanical ventilation (SAR-1000; CWE Inc., Ardmore, PA, USA) was initiated with inhaled gas mixture of oxygen and room air. Ventilation and oxygenation were titrated to maintain end-tidal CO_2_ (ETCO_2_) of 35–45 mm Hg (Capstar CO_2_ analyzer; CWE Inc.) and SpO_2_ of 94–98% (2500A VET; Nonin Medical, Plymouth, MN, USA). Rectal and parietal temperatures were maintained at 37°C ± 1°C using a warming pad (PhysioSuite; Kent Scientific, Torrington, CT, USA). Airway pressure and ECG were also monitored. PE50 catheters were placed into the femoral artery and vein by vascular cutdown technique for arterial blood pressure monitoring, blood withdrawal, and administration of IV fluid and drug. Baseline status was assessed prior to cardiac arrest.

### Cardiac arrest model

Asphyxial cardiac arrest model was performed as described previously with minor modifications [3, 14]. Briefly, rats were chemically paralyzed using IV vecuronium (2 mg/kg over 1 minute). After administration of vecuronium, isoflurane concentration was reduced to 1% and inspired gas was changed to room air to minimize the impact of anesthesia and hyperoxemia on cardiac arrest. At five minutes after vecuronium infusion, asphyxia was induced by stopping the mechanical ventilator. To the best of our knowledge, vecuronium and isoflurane do not affect HSP70 expression in the brain. Cardiac arrest was defined as a mean arterial pressure (MAP) of less than 20 mm Hg, which typically occurred within 3–4 minutes. After 8 minutes of total asphyxia, mechanical ventilation was reinitiated using 100% inspired oxygen. External chest compressions were performed at a rate of 250 beats per minutes using a custom-made pneumatic thumper (S1 Fig). IV epinephrine (0.01 mg/kg) and sodium bicarbonate (1 mEq/kg) were administered at one minute after initiating cardiopulmonary resuscitation. They were repeated every three minutes. If return of spontaneous circulation (ROSC) was not achieved within 10 minutes, resuscitation was terminated. Even if the animal achieve ROSC, early euthanasia was performed based on criteria that universally predict death in this model. These include loss of corneal reflex, heart rate less than 100 beats per minute, SpO_2_ less than 80% for more than 1 hour, and a sustained respiratory rate less than 10 breaths per minute. Rats without ROSC or euthanization within 2 hours after ROSC were considered out of protocol and excluded from analysis. Following ROSC, ventilation and oxygenation were titrated to maintain ETCO_2_ of 35–45 mm Hg and SpO_2_ of 94–98%. After a 30-minute stabilization period, an intra-peritoneal telemetric temperature probe (Physiotel CTA-F40; DSI, St. Paul, MN, USA) was implanted in the peritoneal cavity for wireless monitoring of temperature and ECG. A total of 20 mL/kg 0.9% saline was infused intravenously during the first hour after ROSC. At two hours after ROSC, post-cardiac arrest status of each rat was assessed. Thereafter, rats were de-instrumented, weaned from mechanical ventilation, and extubated. Pulse oximetry was monitored for one hour after extubation. Supplemental oxygen was given by facemask as needed.

### Study protocol

**Fig 1:**
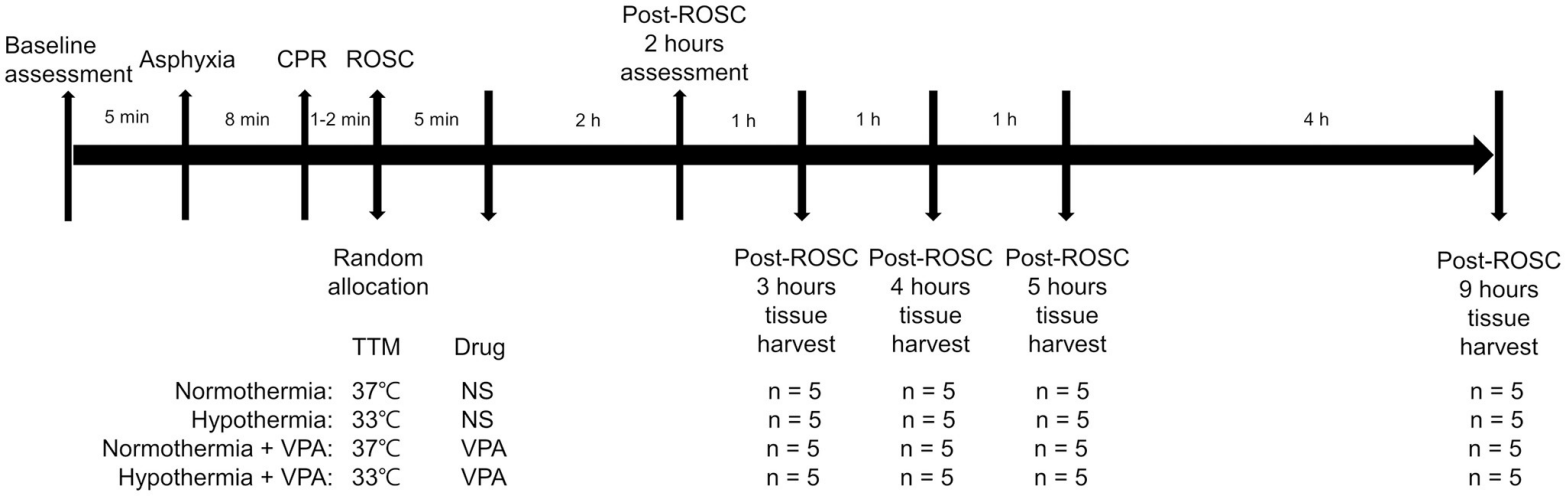
Timeline of experiments. Rats were randomly assigned to four treatment groups after ROSC. Brain tissues were harvested at 3, 4, 5, and 9 hours after ROSC.

Rats resuscitated from an asphyxial cardiac arrest were randomized using a smart phone randomization application (Randomizer for Clinical Trial; Medsharing, Paris, France) to four groups: normothermia (37°C ± 1°C), hypothermia (33°C ± 1°C), normothermia + VPA (300 mg/kg IV initiated 10 min post ROSC and infused over 20 min), and hypothermia + VPA (Fig 1). Either normothermia or hypothermia was initiated immediately after ROSC. A custom-made fan heater designed to regulate temperature by feedback algorithm and a manual water spray were used for hypothermia induction and TTM while rats were under mechanical ventilation (S2 Fig). After weaning of the ventilator, rats were then placed in a custom-made TTM device that could wirelessly monitor and record temperature and ECG signals from the implanted telemetry probe (S3 Fig). The temperature was regulated via software-driven relays connected to a heating coil, water misters, and a cooling fan modified from prior experiments [3, 15] (S4 Fig). VPA at 300 mg/kg or an equivalent volume of saline was intravenously infused over 20 minutes, beginning at 5 minutes after ROSC. In a prior study, histone acetylation was detected at 2 hours after VPA administration [16]. In addition, peak accumulation of HSP70 mRNA and protein appeared at about 3 hours after insult [17]. Activation of caspase-9, a terminal event of intrinsic apoptosis pathway, occurred at 8 hours after cerebral ischemia [18]. However, the peak timing of molecular events can be variable depending on the type of stress, tissue, and experimental settings. Therefore, we performed pilot studies to determine the optimal time at which the activity of target molecules would peak. As a result, histone acetylation was observed to be the best at 3 hours after ROSC, while HSP70 expression and activation of caspase-9 were detected the best at 4–5 hours after ROSC. To present changes over time, we collected regional brain tissues were collected at 3, 4, 5, and 9 hours after ROSC for biochemical analysis.

### Western blots

Ipsilateral hippocampus and cerebellar hemivermis were obtained and homogenized in an ice cold extraction buffer (FNN0011; Invitrogen, Carlsbad, CA, USA) supplemented with protease inhibitor (P2714; Sigma, St. Louis, MO, USA), 5 mM sodium butyrate (303410; Sigma), and 1 mM phenylmethylsulfonyl fluoride (P7676; Sigma) using a tissue homogenizer (TissueLyser II, QIAGEN, Hilden, Germany). Homogenates were centrifuged at 13,000 rpm for 20 minutes. Proteins were then subjected to sodium dodecyl sulfate-polyacrylamide gel (30% acrylamide/bis solution, 37.5:1, #1610158; Bio-Rad, Hercules, CA, USA) electrophoresis. After transferring proteins to nitrocellulose membranes, these membranes were blocked with skim milk and then incubated with the following primary antibodies: anti-histone H3 (#9715, 1:1000; CST, Danvers, MA, USA), anti-acetyl histone H3 (#07–352, 1:1000; Millipore, Billerca, MA, USA), anti-HSP70 (#SC-024, 1:500; Santa Cruz Biotechnology, Dallas, TX, USA), anti-caspase-9 (#9508, 1:1000; CST), and anti-β-actin (#A5441, 1:5000; Sigma). Membranes were then incubated with horseradish peroxidase-linked IgG secondary antibodies (Bio-Rad). Signals were quantified with an image analysis software (CS analyzer 3.0; ATTO Corp., Osaka, Japan). HSP70 signals were expressed relative to β-actin. Acetyl-histone H3 signals were expressed relative to total histone H3. Cleaved caspase-9 40 kDa subunit (p40) signals were expressed relative to procaspase-9. Thereafter, the relative intensity ratio was expressed compared to the corresponding value of the naïve group (n = 5).

### RT-qPCR

Total RNAs were isolated from homogenates of ipsilateral hippocampus and cerebellar hemivermis using TRIzol reagent (Invitrogen) and treated with DNase I (Invitrogen). The quantity of each RNA sample was assessed using a spectrophotometer (NanoDrop One^c^; Thermo Scientific, Waltham, MA). Total RNA (2 μg) was then reverse transcribed using a reverse transcription kit (Promega, Madison, WI, USA) on a T100^™^ thermal cycler (Bio-Rad). The following primers were used: HSP70: 5′-CAGTCGGACATGAAGCACTG-3′ (sense) and 5′-CTCGGCGATCTCCTTCATCT-3′ (antisense); β-actin: 5′-CCTCTATGCCAACACAGTGC-3′ (sense) and 5′-CCTGCTTGCTGATCCACATC-3′ (antisense). Quantitative real-time polymerase chain reactions (RT-qPCR) were performed using a GoTaq^®^ qPCR master mix (Promega) on a CFX96 Real-Time PCR Detection System (Bio-Rad) with the following steps: GoTaq^®^ Hot Start Polymerase activation at 95°C for 2 min, 38 amplification cycles of denaturation at 95°C for 15 seconds followed by annealing and extension at 57°C for 1 minute. HSP70 mRNA expression levels were calculated in fold change and expressed relative to the mean value of the naïve group (n = 5). β-actin was used as an internal control.

### Statistical analysis

Physiologic and hemodynamic data are expressed as mean ± standard deviation (SD). Results of Western blot and RT-qPCR analyses are presented as mean ± standard error of mean. Intergroup comparison was performed by one-way analysis of variances (ANOVA) with Scheffé post-hoc analysis. Biochemical naïve group was excluded from analysis. Two-tailed *p* values of less than 0.05 were considered significant.

## Results

### Animal characteristics

Of a total of 118 rats subjected to cardiac arrest, ROSC was achieved for 100 rats (84.7%). Of these 100 rats with ROSC, 20 were euthanized within 2 hours. Thus, 80 rats were included in the analysis. Five rats were randomly assigned to four groups with different observation periods (3, 4, 5, and 9 hours). A total of 80 rats were assigned to 16 groups. No-flow time (time from MAP < 20 mm Hg to ROSC), number of epinephrine administration, parietal temperature at ROSC, hypothermia induction time (time to reach 33°C), and duration of anesthesia were similar among groups. Although p value of weight obtained by ANOVA was 0.033, post-hoc tests did not show significant intergroup difference (Table 1).

**Table 1:** Comparison of experimental variables between groups.

Experimental variables	Total (n = 80)	Normothermia (n = 20)	Hypothermia (n = 20)	Normothermia + VPA (n = 20)	Hypothermia + VPA (n = 20)	P
Weight (g)	321.73±20.16	326.9±23.52	326.9±19.78	310.8±14.17	322.4±19.03	0.033
No-flow time (sec)	395.66±74.8	390.85±64.72	379.7±32.55	429.25±124.88	382.65±27.43	0.131
Number of epinephrine administration (n)	1±0.66	1.05±0.22	1.25±1.12	1.3±0.66	1.05±0.22	0.511
Parietal temperature at ROSC (°C)	36.23±0.4	36.07±0.46	36.24±0.46	36.39±0.32	36.27±0.29	0.089
Time to reach 33°C	12.31±4.08	n/a	12.25±3.48	n/a	12.37±4.73	0.929
Duration of anesthesia (min)	72.59±17.46	69.05±20.14	74.7±15.71	73.5±17.35	73.1±17.16	0.763

Values are expressed as mean ± SD.

P values were calculated by one-way analysis of variances.

### Hemodynamics and blood gas analysis

**Fig 2:**
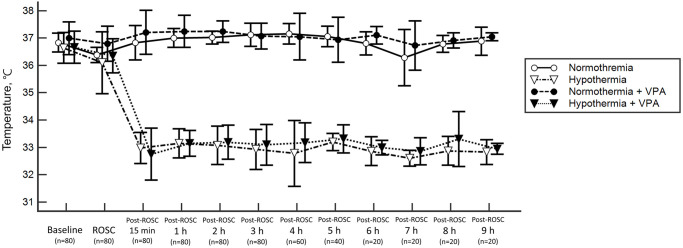
Experimental temperature control. Temporal temperature changes in four treatment groups.

Baseline hemodynamic variables were similar among groups. At two hours after ROSC, heart rate of the hypothermia + VPA group was significantly higher than that of a normothermic group (normothermia and normothermia + VPA). Similarly, MAP of the hypothermia + VPA group was significantly higher than that of a normothermia group. The HCO_3_ levels of hypothermia + VPA group was significantly higher than non-VPA-administered groups (normothermia, hypothermia). The normothermia + VPA group had significantly increased lactate level than the hypothermia group. Although SaO_2_ at 2 hours post-ROSC showed intergroup differences, all groups showed SaO_2_ above 97% (Table 2). Targeted temperature management over time is presented in Fig 2.

**Table 2:** Hemodynamic variables blood gas analysis of baseline and 2 hours after return of spontaneous circulation.

Hemodynamic variables and blood gas analysis	Total (n = 80)	Normothermia (n = 20)	Hypothermia (n = 20)	Normothermia + VPA (n = 20)	Hypothermia + VPA (n = 20)	P
Baseline						
Heart rate (beats per minute)	378.93±35.57	383.95±32.26	363.2±31.38	384.95±44.71	383.6±29.83	0.155
Mean arterial pressure (mm Hg)	90.36±9.99	93.67±6.77	87.62±11.25	89.22±6.41	90.93±13.41	0.261
End tidal CO_2_ (%)	41.89±2.01	42.1±2.05	41.6±2.54	41.95±1.88	41.9±1.59	0.889
Arterial pH	7.37±0.03	7.37±0.02	7.36±0.03	7.36±0.29	7.37±0.03	0.686
PaCO_2_ (mm Hg)	35.95±6.23	36.5±7.35	33.63±6.45	36.81±5.56	38.39±4.78	0.106
PaO_2_ (mm Hg)	169.76±31.67	171.16±35.57	168±30.45	162.58±35.15	177±25.62	0.556
HCO_3_ (mmol/L)	20.97±3.59	21.23±3.75	19.35±4.1	21.12±3.61	22.21±2.28	0.084
SaO_2_ (%)	99.41±0.81	99.42±0.84	99.3±0.8	99.32±1	99.6±0.6	0.641
Lactate (mmol/L)	0.68±0.34	0.65±0.37	0.69±0.37	0.68±0.35	0.69±0.3	0.987
Post-ROSC 2 hours						
Parietal temperature (°C)	35.24±2.06	37.23±0.35^b^^d^	33.02±0.45^a^^c^^d^	37.02±0.34^b^^d^	33.45±0.44^a^^b^^c^	<0.001
Heart rate (beats per minute)	351.39±54.03	333.8±49.92^d^	349.2±61.48	333±36.61^d^	389.55±48.02^a^^c^	<0.001
Mean arterial pressure (mm Hg)	107.03±22.09	97.18±25.12^d^	113.63±17.43	100.75±23.92	116.53±15.41^a^	0.009
End tidal CO_2_ (%)	42.02±2.59	41.18±2.41	41.95±3.36	42.35±2.66	42.6±1.57	0.329
Arterial pH	7.28±0.05	7.28±0.05	7.27±0.06	7.28±0.05	7.27±0.06	0.854
PaCO_2_ (mm Hg)	39.82±8.9	36.68±7.97	37.33±10.37	41.31±8.07	43.67±7.6	0.04
PaO_2_ (mm Hg)	139.19±68.15	130.44±75.67	162.4±68.32	108.15±25.06	154.9±83	0.046
HCO_3_ (mmol/L)	19.12±3.78	17.56±3.83^d^	17.9±3.97^d^	19.51±2.56	21.37±3.61^a^^b^	0.004
SaO_2_ (%)	97.97±1.66	97.5±1.89^b^	98.9±1.12^a^^c^	96.9±1.74^b^^d^	98.55±1^c^	<0.001
Lactate (mmol/L)	1.04±0.95	0.79±0.8	0.62±0.52^c^	1.51±1.46^b^	1.22±0.42	0.011

Values are expressed as mean ± SD.

P values were calculated by one-way analysis of variances.

^a^Significantly different compared to the normothermia group by post-hoc Scheffé test (P<0.05).

^b^Significantly different compared to the hypothermia group by post-hoc Scheffé test (P<0.05).

^c^Significantly different compared to the normothermia + VPA group by post-hoc Scheffé test (P<0.05).

^d^Significantly different compared to the hypothermia + VPA group by post-hoc Scheffé test (P<0.05).

### Acetylated histone H3

**Fig 3:**
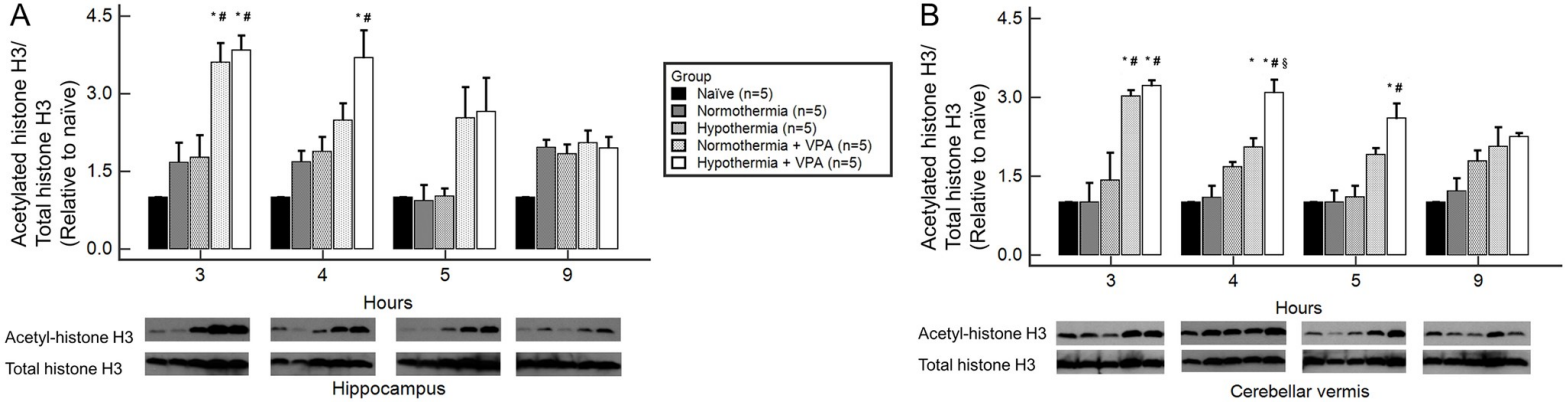
Acetylated histone H3 of regional brain homogenates obtained at 3, 4, 5, and 9 hours after ROSC by Western blot analysis. At three hours after ROSC, VPA-administered groups (normothermia + VPA, hypothermia + VPA) showed significantly higher levels of acetylated histone H3 level than non-VPA-administered groups both in the hippocampus (A) and the cerebellar vermis (B). Bars represent mean ± SEM. Intergroup comparison was performed by one-way analysis of variances with Scheffé post-hoc analysis. *, *p* < 0.05 versus normothermia; ^#^, *p* < 0.05 versus hypothermia; ^§^, *p* < 0.05 versus normothermia + VPA.

At three hours after ROSC, VPA-administered groups (normothermia + VPA, hypothermia + VPA) showed significantly higher levels of acetylated histone H3 than non-VPA-administered groups. At four hours after ROSC, the hypothermia + VPA group showed significantly higher acetylated histone H3 levels than non-VPA-administered groups. At five hours after ROSC, the hypothermia + VPA group showed significantly higher acetylated histone H3 levels than non-VPA-administered groups only in the cerebellar vermis. In summary, VPA administration induced acetylation of histone H3 regardless of the temperature or the region at 3 hours after ROSC. Afterwards, histone acetylation showed a trend of decreasing over time (Fig 3).

### HSP70

**Fig 4:**
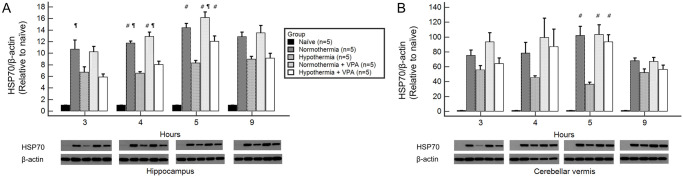
HSP70 mRNA of regional brain homogenates obtained at 3, 4, 5, and 9 hours after ROSC by RT-qPCR analysis. At four hours after ROSC, normothermic groups (normothermia, normothermia + VPA) showed significantly higher HSP70 mRNA level than the hypothermia group regardless of regions. The hypothermia + VPA group showed significantly higher HSP70 mRNA level in the cerebellar vermis than the hypothermia group at 4 hours after ROSC (B). In the hippocampus, the hypothermia + VPA group also showed higher HSP70 mRNA level than the hypothermia group at 4 hours after ROSC, although such difference was not statistically significant (A). Bars represent mean ± SEM. Intergroup comparison was performed by one-way analysis of variances with Scheffé post-hoc analysis. ^#^, *p* < 0.05 versus hypothermia; ^¶^, *p* < 0.05 versus hypothermia + VPA.

At four hours after ROSC, HSP70 mRNA expression levels of normothermic groups were significantly higher than that of the hypothermia group in both regions (Fig 4). The hypothermia + VPA group showed significantly higher HSP70 mRNA levels in the cerebellar vermis than the hypothermia group at 4 hours after ROSC. In the hippocampus, the hypothermia + VPA group showed higher HSP70 mRNA expression compared to the hypothermia group at 4 hours after ROSC, although the difference between the two was not statistically significant. In general, normothermia and VPA upregulated HSP70 mRNA expression, while hypothermia attenuated HSP70 mRNA expression. However, VPA treatment combined with hypothermia significantly upregulated HSP70 mRNA expression compared to a single hypothermia treatment in the cerebellar vermis.

**Fig 5:**
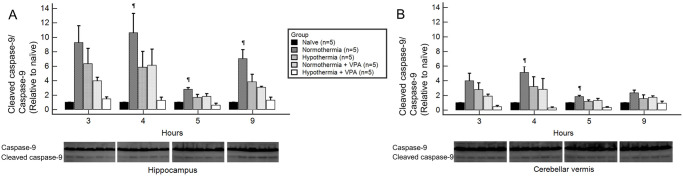
HSP70 of regional brain homogenates obtained at 3, 4, 5, and 9 hours after ROSC by Western blot analysis. At five hours after ROSC, the normothermic group showed significantly higher HSP70 levels than the hypotherma group both in the hippocampus (A) and the cerebellar vermis (B). HSP70 expression was significantly higher in the hypothermia + VPA group than in the hypothermia group in both regions. Bars represent mean ± SEM. Intergroup comparison was performed by one-way analysis of variances with Scheffé post-hoc analysis. ^#^, *p* < 0.05 versus hypothermia; ^¶^, *p* < 0.05 versus hypothermia + VPA.

Similarly, HSP70 protein levels were higher in normothermic groups than in the hypothermia group at 5 hours after ROSC regardless of regions (Fig 5). The hypothermia + VPA group had significantly higher HSP70 protein level than the hypothermia group at 5 hours after ROSC regardless of regions. To summarize, a single hypothermia treatment attenuated HSP70 expression. However, hypothermia treatment combined with VPA administration significantly increased HSP70 mRNA at 4 hours after ROSC and HSP70 protein at 5 hours after ROSC compared to the hypothermia treatment alone.

### Cleaved caspase-9

**Fig 6:**
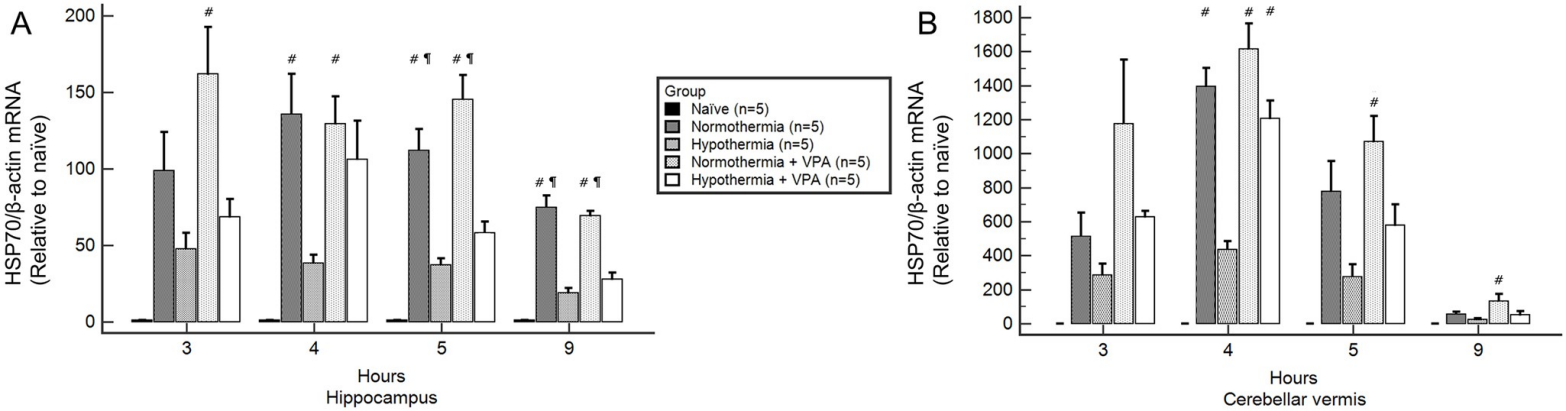
Cleaved caspase-9 of regional brain homogenates obtained at 3, 4, 5, and 9 hours after ROSC by Western blot analysis. At four and five hours after ROSC, only the hypothermia + VPA group showed significantly decreased cleaved caspase-9 levels compared to the normothermia group both in the hippocampus (A) and the cerebellar vermis (B). In the hippocampus, cleaved caspase-9 level in the hypothermia + VPA group was significantly lower than that in the normothermia group until 9 hours after ROSC (B). Bars represent mean ± SEM. Intergroup comparison was performed by one-way analysis of variances with Scheffé post-hoc analysis. ^¶^, *p* < 0.05 versus hypothermia + VPA.

At four and five hours after ROSC, only the hypothermia + VPA group demonstrated significantly lower cleaved caspase-9 levels than the normothermia group regardless of regions (Fig 6). In the hippocampus, cleaved caspase-9 level in the hypothermia + VPA group remained significantly lower than the normothermia group until 9 hours after ROSC.

## Discussion

This study demonstrated that combined treatment of hypothermia with VPA significantly upregulated HSP70 level compared to hypothermia alone. Intergroup comparison revealed that the hypothermia + VPA group had significant attenuation of caspase-9 activation than the normothermia group. Single treatment groups (hypothermia, normothermia + VPA) did not show significantly reduced caspase-9 activation compared to the normothermia group. However, this is not inconsistent from the prior studies which showed reduced caspase-9 activation by hypothermia. At 5 hours after cardiac arrest, comparison between just two groups by t-test showed significantly reduced caspase-9 activity of the hypothermia group compared to the normothermia group regardless of region (hippocampus, *p* = 0.048; cerebellar vermis, *p* = 0.049). The hypothermia + VPA group showed significantly increased heart rate, MAP, and HCO_3_^-^ levels compared to the normothermia group.

Although the therapeutic value of hypothermic TTM at clinical level is a subject of debate, hypothermia remains an important neuroprotective strategy. Neuroprotective mechanisms of hypothermia have not been completely elucidated yet. However, multiple experimental and clinical studies have revealed that downregulation of cellular metabolism seems to be the main neuroprotective mechanism of hypothermia [19]. Hypothermia also inhibits excitotoxicity by limiting influx of toxic levels of Ca^2+^ [20]. Many studies have shown that hypothermia can reduce reactive oxygen species and inhibit nuclear factor kappa B (NFkB) [21, 22]. Hypothermia seems to influence apoptotic and survival signaling pathways by affecting multiple molecules such as BCL-2 family members, protein kinase, cytochrome c, phosphatase and tensin homolog, Fas ligand, and apoptosis-inducing factor (AIF) [23–27].

HSP70 is a neuroprotective molecular chaperone. It blocks the assembly of a functional apoptosome by interfering with the recruitment of procaspase-9 to apoptotic protease activating factor-1, which in turn inhibits caspase-dependent apoptotic pathways [28, 29]. Other proposed neuroprotective mechanisms of HSP70 include suppressing c-Jun N-terminal kinase, decreasing NFkB activation, and blocking AIF [30–32]. Currently, it is unclear whether hypothermia affects HSP70 expression. Many studies have reported hypothermia downregulates HSP70, while a few studies have reported that hypothermia upregulates HSP70 [7–13, 33–37]. There are several possible reasons for such discrepancy. First, hypothermia itself can denature protein. Thus, cold-induced cell damage may lead to heat shock-like response. In several studies demonstrating hypothermia-induced upregulation of HSP70, other stresses such as oxygen-glucose deprivation or ischemia were not applied [33, 35]. Therefore, heat shock-like response might have been induced by hypothermia itself. Second, adrenergic receptor ligands have a major impact on HSP70 expression [35]. In *in vivo* experiments, hypothermia may facilitate norepinephrine release and activate adrenergic receptors, leading to upregulation of HSP70 [34–36]. In addition, according to our experiences, body temperatures of rats spontaneously drop after injury. To maintain normothermia in injured rats for prolonged duration, active heating is mandatory. A recent study has shown temperature drops of post-cardiac arrest rats after prolonged duration (7 hours) of controlled normothermia, consistent with our observations [38]. Meanwhile, an animal study has reported hypothermia-induced upregulation of HSP70 without using an active heating device [36]. Leaving injured rats under room temperature without active heating may lead to mild hypothermia. Thus, comparison between hypothermic rats under cold-stressed condition and subnormothermic rats in the normothermia group might have skewed results. Third, expression of HSP70 requires active cell metabolism. Heat shock response can be activated only after reperfusion [33]. However, a recent *in vitro* study has shown hypothermia-induced upregulation of HSP70 during oxygen-glucose deprivation period [37]. In this study, hypothermia-induced HSP70 upregulation did not occur at the post-reperfusion period. Thus, hypothermia during ischemic period might have paradoxically enhanced HSP70 expression by maintaining minimal metabolic requirements such as ATP production and macromolecule synthesis. Collectively, HSP70 does not seem to have a role in hypothermia-induced neuroprotection [7–11]. Rather, hypothermia seems to attenuate HSP70-mediated anti-apoptotic pathway. Facilitating HSP70 expression can be a novel strategy to compensate for decreased anti-apoptotic activity of HSP70 under hypothermia. To induce HSP70 expression, many studies have used genetic mutation animal models, gene transfer, or heat stress. However, these methods are impractical at clinical level. Therefore, many pharmacological compounds have been introduced for HSP70 induction [39]. VPA is one pharmacologic agent known to inhibit class I HDACs to induce hyperacetylation of histone and activation of HSP70 promoter [40, 41]. Recently, VPA has emerged as an anti-cancer drug due to its epigenetic regulation of expression of genes involving in cell cycle arrest, angiogenesis, metastasis, differentiation, and senescence [42]. Epigenetic mechanisms of VPA have beneficial effects on epilepsy and malignancies. They are also responsible for more general neuroprotective mechanisms [43]. VPA has been demonstrated to be neuroprotective in many models of neuronal injury [4, 6, 43]. Several animal studies have demonstrated neuroprotective properties of VPA after cardiac arrest [1–3, 44]. Therefore, combination of hypothermia and VPA may yield better results. A few *in vitro* studies have reported enhanced neuroprotective effects of combined treatment of hypothermia and VPA [45, 46]. Moreover, two separate *in vivo* studies have shown improved survival and outcomes after combination therapy of hypothermia and VPA in a rat model of asphyxial cardiac arrest [2, 3]. In addition, combination of hypothermia and VPA shows neuroprotective benefit in a canine model of hypothermic circulatory arrest [47]. However, additive or synergistic neuroprotective mechanism of VPA combined with hypothermia has not been reported yet. In the present study, hyperacetylation of histone was observed in the VPA-administered group, which in turn upregulated HSP70 at transcriptional and protein levels. It should be noted that HSP70 was significantly more expressed in the hypothermia + VPA group compared to that in the hypothermia group. VPA administration induced heat shock-like response even under hypothermia. Consequently, only the hypothermia + VPA group showed significantly attenuated caspase-dependent apoptosis compared to the normothermia group. Our results suggest that epigenetic modification of histone through inhibition of HDAC by VPA can lead to reinforcement of HSP70-mediated anti-apoptotic signaling pathway which is suppressed under hypothermia.

### Study limitations

First, VPA-administered groups showed higher lactate levels than non-VPA-administered groups, consistent with prior studies [3, 44]. VPA is known to increase lactate level by partially inhibiting gluconeogenesis or pyruvate oxidation, which may lead to hepatotoxicity [48]. Maximal neuroprotective dose of VPA with minimal adverse effects should be determined in future studies. A recent study has shown that VPA up to 150 mg/kg can be safely tolerated in a swine cardiac arrest model [44]. However, in the present study, a high lactate level of 300mg/kg in a rat model was not accompanied by acidosis or hypotension. It rather improved hemodynamics (Table 1). Second, both hypothermia and HSP70-mediated neuroprotection involve multiple mechanisms. Thus, something else yet unidentified might also contribute to the combined neuroprotective mechanism of hypothermia + VPA. Inhibition of NFkB and AIF might be such unidentified mechanisms as they are commonly involved in hypothermia and HSP70-mediated neuroprotection [22, 27, 31, 32]. Thus, they might exert additive or synergistic effect. Further studies are required to better understand detailed signaling mechanisms involved in the combined neuroprotective effect as current evidences are insufficient to explain many aspects of the combination treatment with hypothermia and VPA.

## Conclusions

In summary, hypothermia attenuates expression of HSP70 at transcriptional level. However, epigenetic upregulation of HSP70 by HDAC-inhibiting effect of VPA can reinforce HSP70-mediated anti-apoptotic activity suppressed by hypothermia. Present data suggest that VPA could be used as a neuroprotective drug in combination with hypothermia to compensate for hypothermia-related HSP70 suppression.

## Supporting information

S1 Fig Custom-made pneumatic thumper for rat. (TIF)

S2 Fig Custom-made fan heater with feedback temperature control. (TIF)

S3 Fig Custom-made targeted temperature management device for rat. (TIF)

S4 Fig Chamber equipped with a heating coil, water misters and a cooling fan connected to an exhaust hood. (TIF)

S1 Raw images Original images of the Western blots. The membranes were exposed to film (CP-BU new; AGFA, Mortsel, Belgium) and scanned by a film scanner (Perfection 4180 photo, Epson, Suwa, Japan).
(TIF)
