# Supplementary figures and images for: HSP70-mediated neuroprotection by combined treatment of valproic acid with hypothermia in a rat asphyxial cardiac arrest model
Source: PLoS One. 2021 Jun 17;16(6):e0253328. doi: 10.1371/journal.pone.0253328 (PMC8211226; doi:10.1371/journal.pone.0253328)

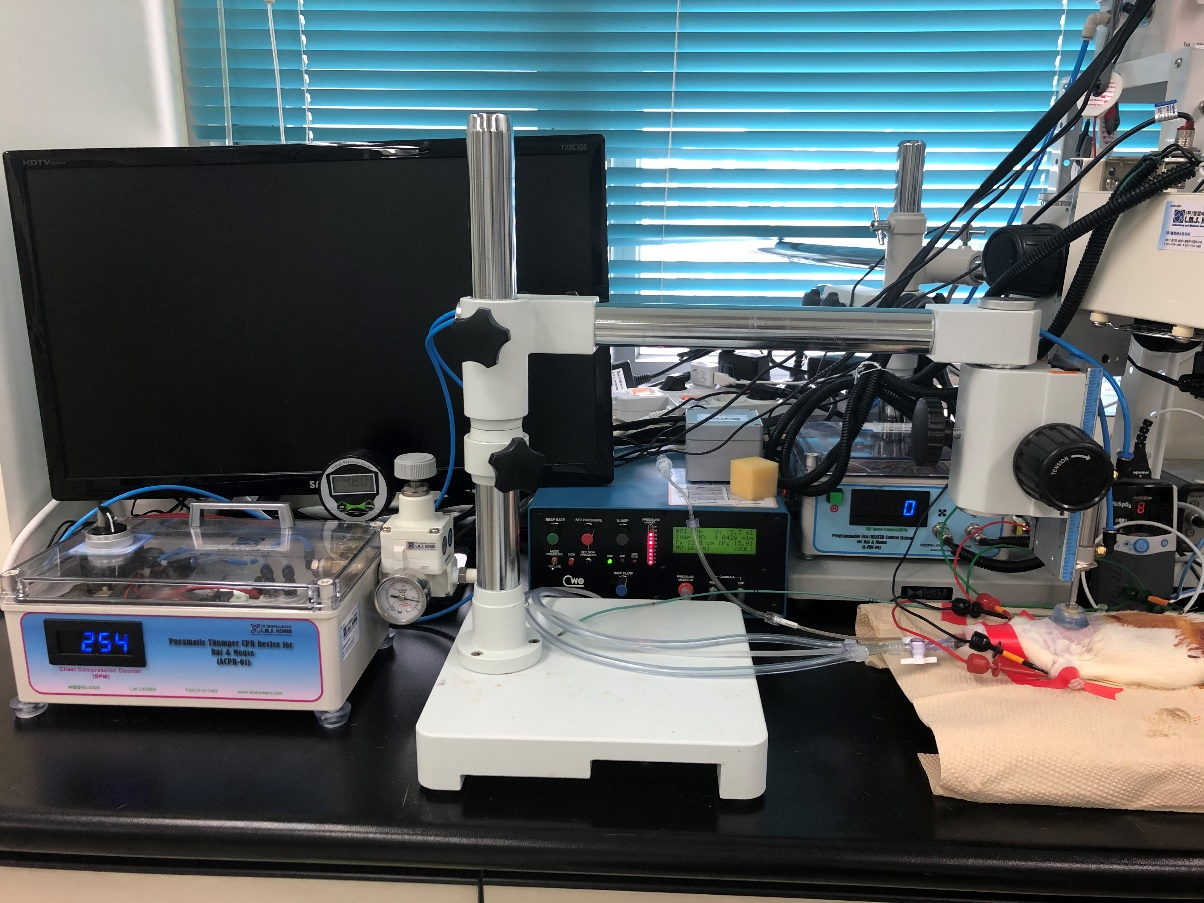

Supplement: S1 Fig — (TIF) [file pone.0253328.s001.tif]

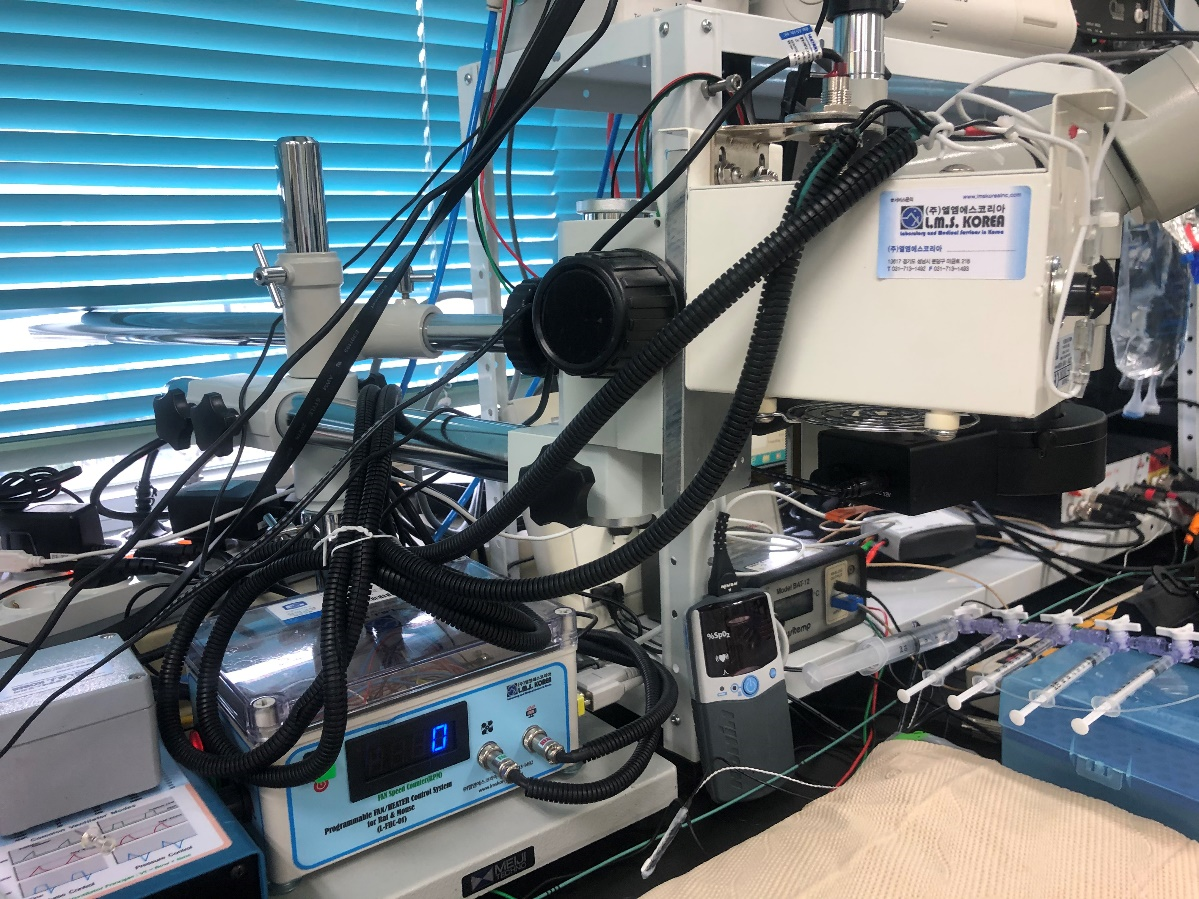

Supplement: S2 Fig — (TIF) [file pone.0253328.s002.tif]

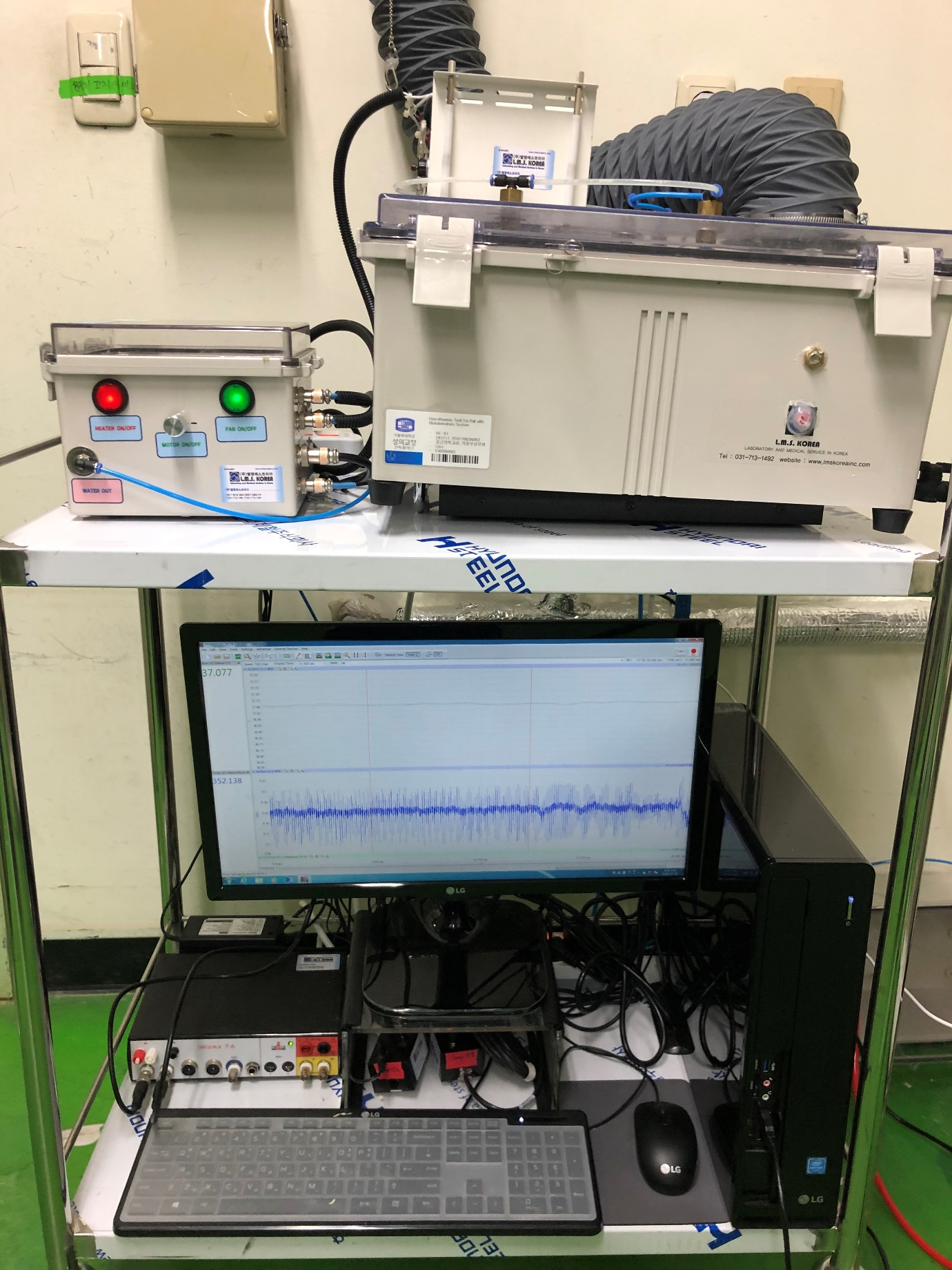

Supplement: S3 Fig — (TIF) [file pone.0253328.s003.tif]

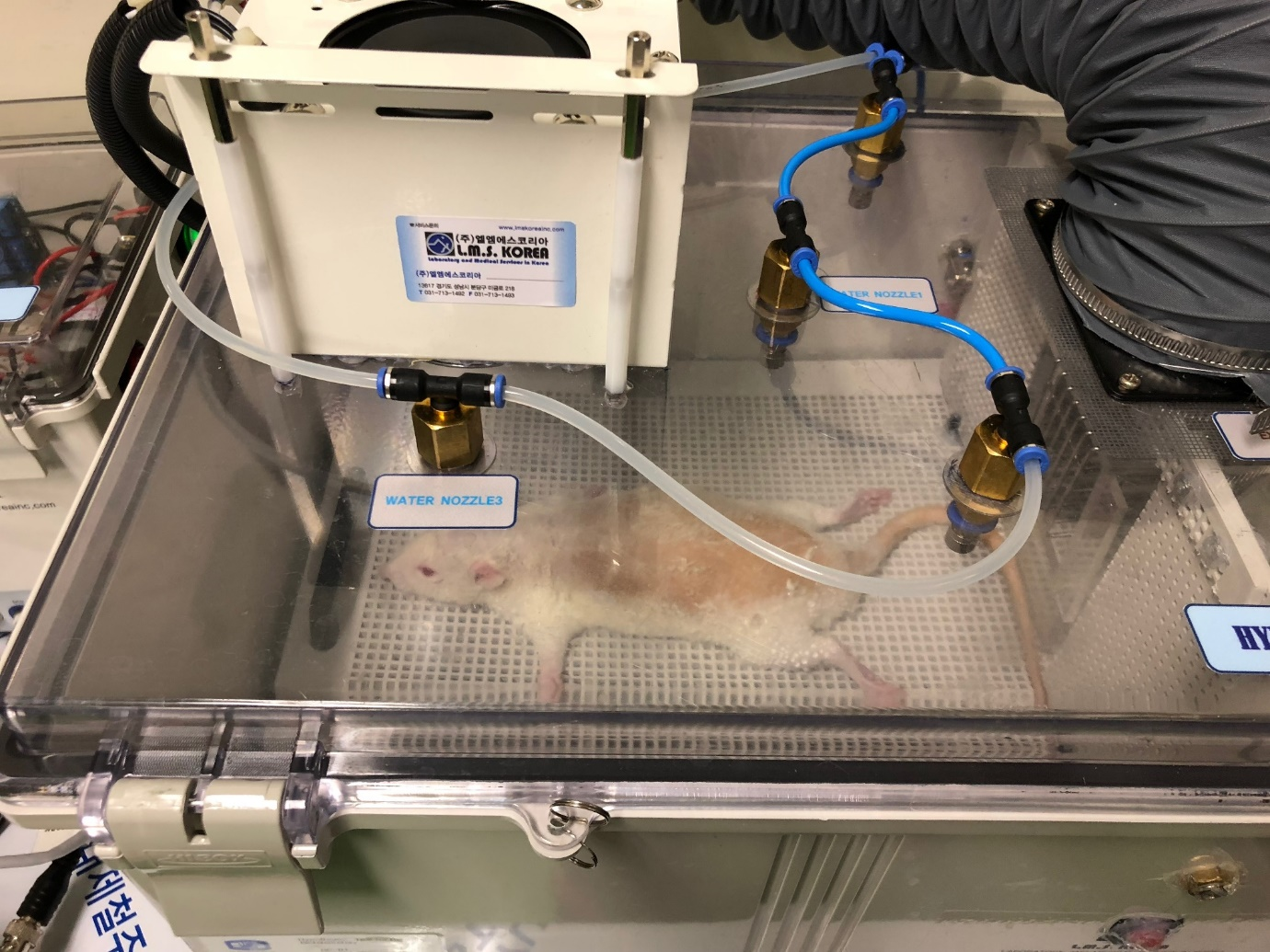

Supplement: S4 Fig — (TIF) [file pone.0253328.s004.tif]

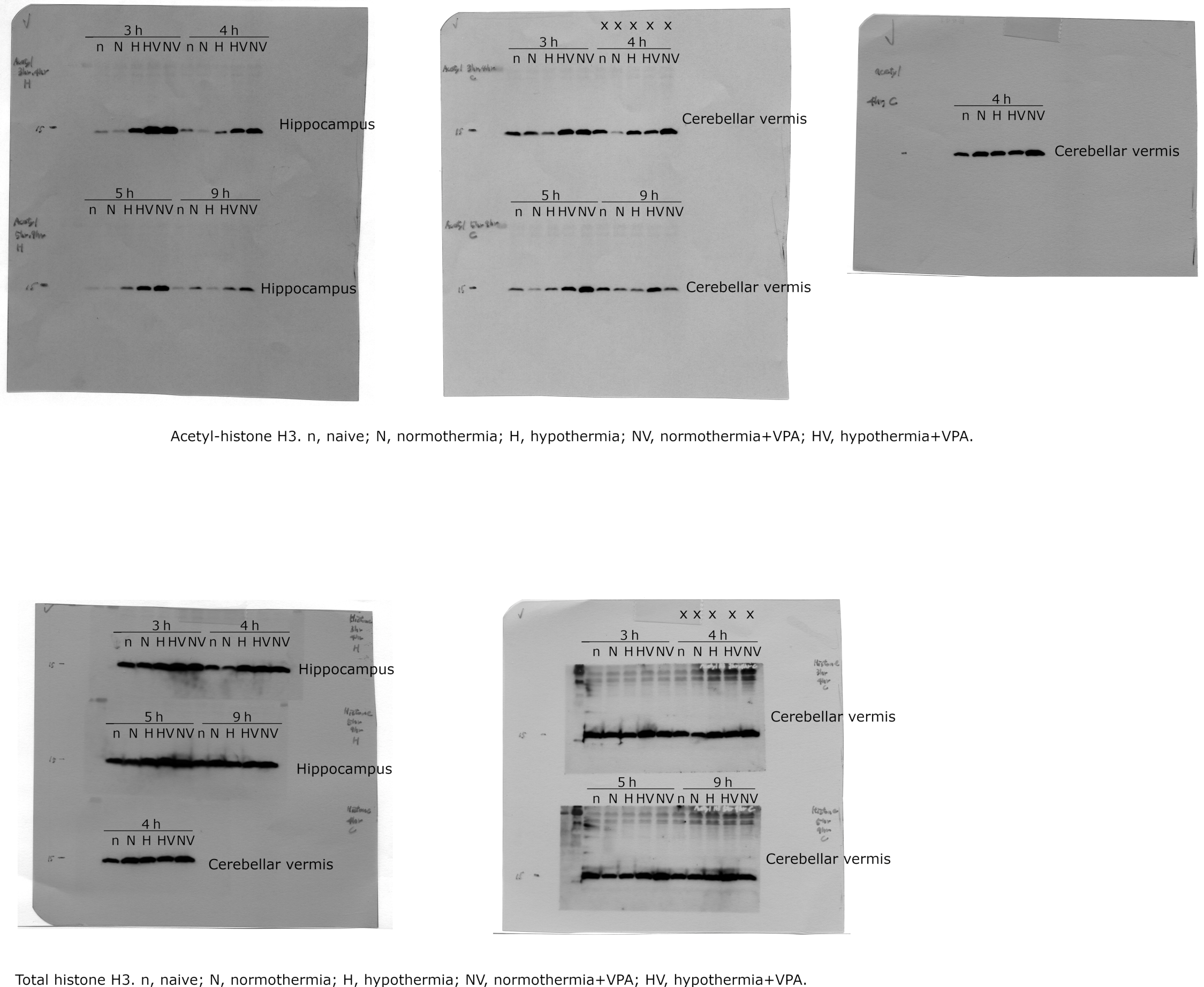

Supplement: S1 Raw images — The membranes were exposed to film (CP-BU new; AGFA, Mortsel, Belgium) and scanned by a film scanner (Perfection 4180 photo, Epson, Suwa, Japan). (TIF) [file pone.0253328.s005.tif]
